# Ciclopirox olamine induces ferritinophagy and reduces cyst burden in polycystic kidney disease

**DOI:** 10.1172/jci.insight.141299

**Published:** 2021-03-30

**Authors:** Priyanka S. Radadiya, Mackenzie M. Thornton, Rajni V. Puri, Sireesha Yerrathota, Johnny Dinh-Phan, Brenda Magenheimer, Dharmalingam Subramaniam, Pamela V. Tran, Hao Zhu, Subhashini Bolisetty, James P. Calvet, Darren P. Wallace, Madhulika Sharma

**Affiliations:** 1 Department of Internal Medicine,; 2 Jared Grantham Kidney Institute,; 3 Department of Biochemistry and Molecular Biology,; 4 Department of Cancer Biology,; 5 Department of Anatomy and Cell Biology, and; 6 Department of Clinical Laboratory Sciences, University of Kansas Medical Center, Kansas City, Kansas, USA.; 7 Department of Internal Medicine, School of Medicine, University of Alabama at Birmingham, Alabama, USA.

**Keywords:** Nephrology, Chronic kidney disease

## Abstract

Despite the recent launch of tolvaptan, the search for safer polycystic kidney disease (PKD) drugs continues. Ciclopirox (CPX) or its olamine salt (CPX-O) is contained in a number of commercially available antifungal agents. CPX is also reported to possess anticancer activity. Several mechanisms of action have been proposed, including chelation of iron and inhibition of iron-dependent enzymes. Here, we show that CPX-O inhibited in vitro cystogenesis of primary human PKD cyst-lining epithelial cells cultured in a 3D collagen matrix. To assess the in vivo role of CPX-O, we treated PKD mice with CPX-O. CPX-O reduced the kidney-to-body weight ratios of PKD mice. The CPX-O treatment was also associated with decreased cell proliferation, decreased cystic area, and improved renal function. Ferritin levels were markedly elevated in cystic kidneys of PKD mice, and CPX-O treatment reduced renal ferritin levels. The reduction in ferritin was associated with increased ferritinophagy marker nuclear receptor coactivator 4, which reversed upon CPX-O treatment in PKD mice. Interestingly, these effects on ferritin appeared independent of iron. These data suggest that CPX-O can induce ferritin degradation via ferritinophagy, which is associated with decreased cyst growth progression in PKD mice. Most importantly these data indicate that CPX-O has the potential to treat autosomal dominant PKD.

## Graphical abstract

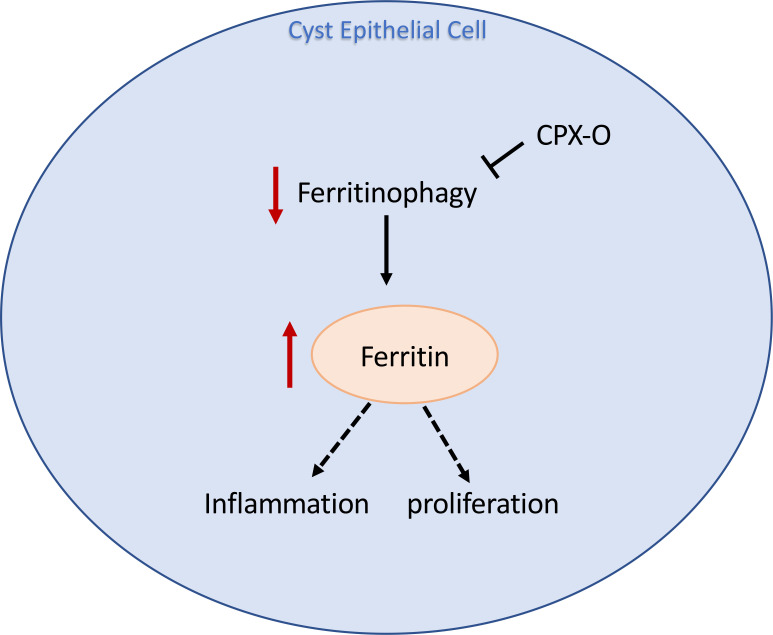

## Introduction

Polycystic kidney disease (PKD) is a genetic disease characterized by multiple cysts in the kidneys, which enlarge over time and lead to end-stage renal disease. The incidence of autosomal dominant polycystic kidney disease (ADPKD) is 1 in every 1000 births. About 85% of ADPKD cases have mutations in polycystin 1 (PC1) and the remaining 15% in polycystin 2 (PC2) (proteins encoded by *PKD1* and *PKD2*, respectively) (1, 2). Both PC1 and PC2 have been found to be localized to cilia. PC1 and PC2 function in the cilia and appear to be important in the regulation of intracellular Ca^2+^. Decreased intracellular Ca^2+^ levels in ADPKD kidney cells in combination with activation of adenylate cyclase and accumulation of cAMP lead to increased proliferation and fluid secretion and contribute to enlargement of cysts (3–6). Many signaling pathways have been shown to be deregulated in response to loss-of-function mutations in PKD (2, 3, 7, 8).

Since there are similarities in cancer progression and PKD (9, 10), we sought drugs in the cancer literature that could be repurposed for PKD. Specifically, we looked for drugs that could safely and efficiently reach the urinary tract, inhibit cell cycle progression, and/or inhibit fluid secretion. Our literature search revealed ciclopirox as a candidate drug that could be effective in PKD. 6-Cyclohexyl-1-hydroxy-4-methyl-2(1*H*)-pyridone (ciclopirox or CPX) or its olamine salt 6-cyclohexyl-1-hydroxy-4-methyl-2(1*H*)-pyridone 2-aminoethanol salt (ciclopirox olamine or CPX-O) has been reported to efficiently reach the urinary tract, spare normal cells, inhibit cell cycle progression, and affect fluid secretion by correcting chloride channel expression (11–14). In addition, CPX inhibits several pathways that are also indicated as targets of therapy in PKD, such as Notch, mTOR, MEK/ERK, and Wnt signaling pathways (13, 15–18).

CPX and specifically CPX-O are antifungal, topical agents used in a variety of formulations, such as gels, creams, and shampoos, for treatment of cutaneous fungal infections (19). However, over the last decade, the wide use of CPX or CPX-O in other diseases has been recognized (20–22). Furthermore, the safe therapeutic response of CPX-O has been tested in various animals, including pigs and dogs (20). Preclinical studies have shown that systemic administration of CPX-O in hematologic malignancies resulted in antitumor effects (23). The proposed mechanisms of CPX include chelation of polyvalent metal cations, such as Fe^3+^, by which it can inhibit intracellular iron-dependent enzymes, such as ribonucleotide reductase and components of hypoxia, Wnt, and Notch signaling (16, 24, 25). CPX is also involved in ferritin degradation protection against ferroptosis (24, 26–28). Ferritin is a hetero-polymeric molecule composed of 24 heavy and light subunits, which can store up to 4500 iron atoms (29). Ferritin is composed of varying proportions of 2 gene products, heavy ferritin (FTH1) and light ferritin (FTL). The FTH1 exhibits ferroxidase activity, which converts toxic ferrous ions into ferric ions, while the FTL subunit facilitates iron storage (30–32). Given the role of CPX or CPX-O as an iron chelator and its role in multiple pathways, we hypothesized that CPX-O can reduce cyst progression in PKD by mechanisms that involve the iron/ferritin axis.

Our data indicate that CPX-O can reduce cyst progression in 3D collagen assays from primary cyst epithelial cells from patients with ADPKD and in a PKD mouse model. Mechanistically, CPX-O reduced ferritin expression in vivo in an iron-independent manner by the process of ferritinophagy, and this process was associated with reduced cyst progression in PKD.

## Results

### CPX-O inhibits cyst progression of ADPKD kidney cells in 3D cyst assays.

**Figure 1:**
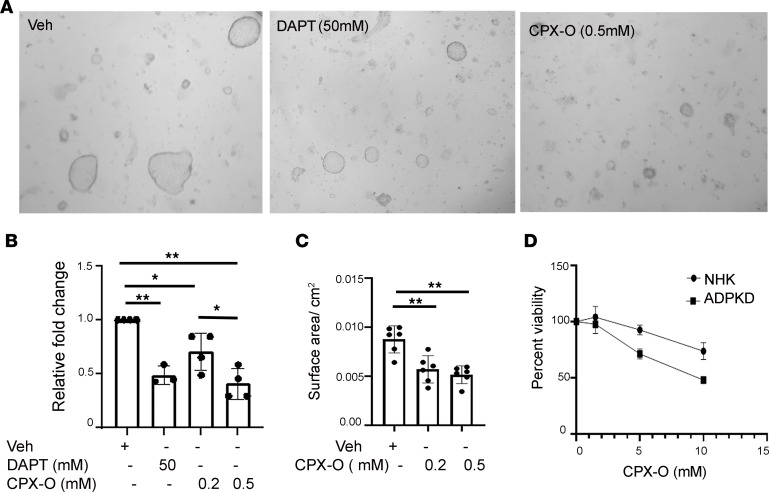
CPX-O inhibits cyst formation of ADPKD cells. ADPKD cells were grown to form cysts on a 3D system using collagen matrix in the presence of FSK and EGF for 3–5 days followed by treatment with vehicle, CPX-O, or N-[N-(3,5-Difluorophenacetyl)-L-alanyl]-S-phenylglycine t-butyl ester (DAPT) for 6 additional days. (**A**) Cysts were fixed, imaged, and measured. Original magnification, ×10. (**B**) Average fold change in cyst size with vehicle cyst size set at 1 (from 4 patients). Within each patient 6 replicates/treatment were used and data were averaged. (**C**) Cyst size is represented as surface area ± SEM from a single ADPKD patient (K298) (6 replicates/treatment). (**D**) Primary cells from normal human kidney (NHK) (*n* = 3) and ADPKD kidney (*n* = 3) cells were cultured in the presence of CPX-O for 6 days followed by viability assays. Data presented as percentage viability ± SEM. Statistical significance was determined using 1-way ANOVA followed by Tukey’s honestly significant difference (HSD) test (*P < 0.05, **P < 0.01).

To first validate whether CPX-O may be a drug of choice for PKD in the clinic, and whether further mouse studies are required to validate it, we performed 3D cyst assays using primary ADPKD renal epithelial cells (33). ADPKD cells were induced to form cysts in a collagen gel in the presence of the cAMP agonist forskolin (FSK) and growth factor EGF. After cyst formation, CPX-O at desired concentrations or vehicle was administered for 6 days, and the effects of CPX-O on cyst progression were evaluated. As shown in Figure 1, A and B, vehicle alone did not affect cyst size, but concentrations as low as 0.2 μM CPX significantly (*P* < 0.05) decreased cyst area and further decreased at 0.5 μM CPX-O (*P* < 0.01). We have previously shown equivalent fold reduction in cyst size with 50 μM DAPT treatment (8). DAPT is a γ-secretase inhibitor widely used to inhibit Notch signaling. Figure 1B represents average fold change of cyst size from 4 patients (in 6 replicates each). In Figure 1C, data from individual patient cells (K268) are shown (in 6 replicates), and cyst size is represented as surface area. A similar effect with increasing CPX-O concentrations as in Figure 1B was seen, indicating that CPX-O inhibits cyst formation in ADPKD. To determine whether decreased cystic area resulted from CPX-O toxicity, cell viability was determined with increasing concentrations of CPX in ADPKD cells as well as in cells obtained from normal human kidney (NHK) over 6 days in culture. As shown (Figure 1D), there was no cell death observed in either NHK or ADPKD cells at a concentration of 0.2 to 0.5 μM CPX-O, whereas at a concentration of 2 μM or above, viability declined in ADPKD cells at a much faster rate as compared with the NHK cells, showing that ADPKD cells were more sensitive to CPX-O than NHK cells.

### CPX-O attenuates disease progression in a mouse model of ADPKD.

**Figure 2:**
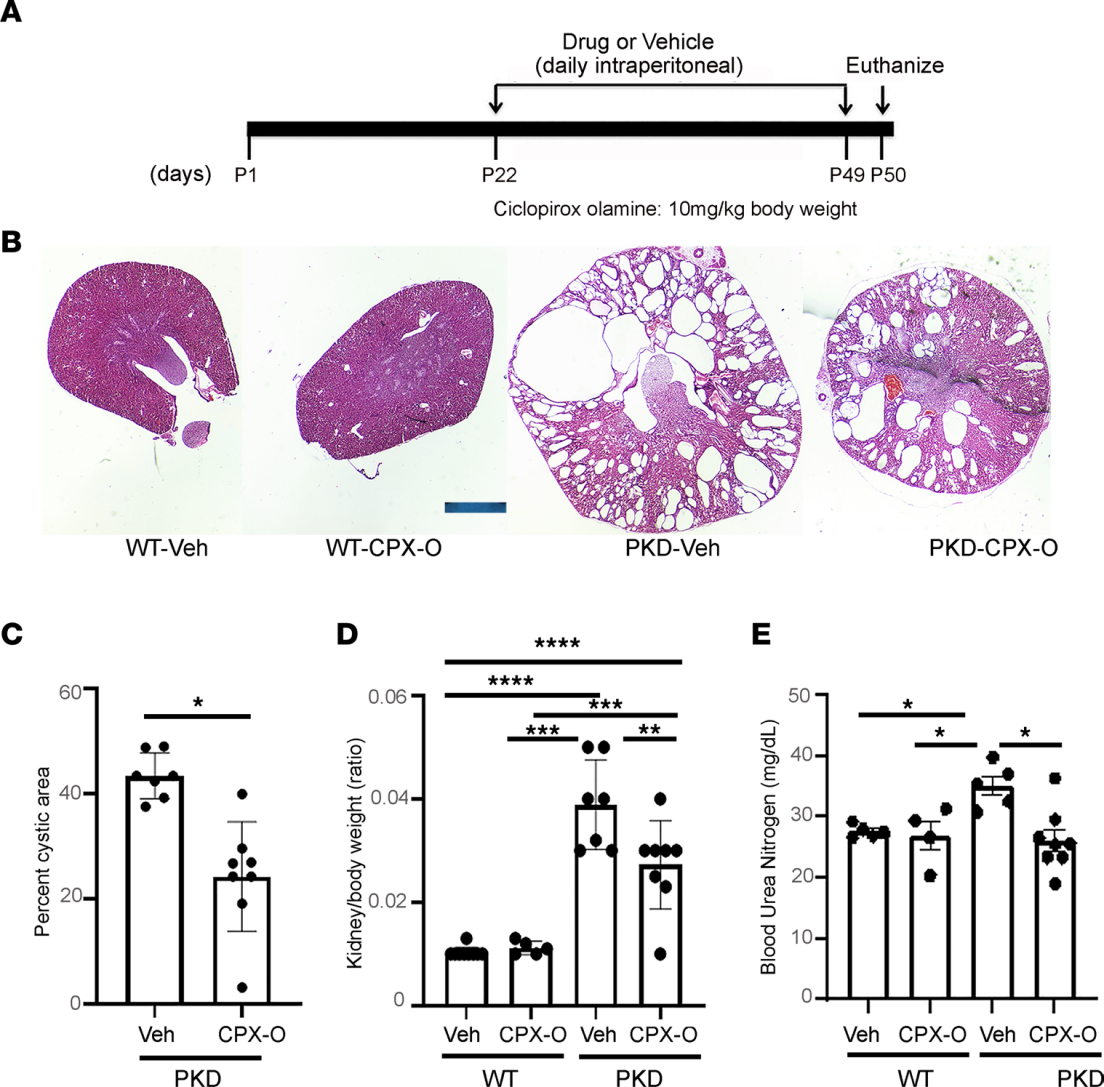
CPX-O ameliorates disease progression in a mouse model of ADPKD. (**A**) Experimental timeline where 22-day-old PKD or WT mice were intraperitoneally injected with vehicle or CPX-O (10 mg/kg body weight) for 27 consecutive days until P49. At P50, mice were euthanized and samples were collected. (**B**) H&E staining of kidney sections. Representative images of each treatment group are shown. (**C**) Renal cystic index of vehicle-treated (*n* = 7) and CPX-O–treated (*n* = 8) PKD mice, presented as percentage cystic area ± SEM. (**D**) Kidney-to-body weight ratio from vehicle- or CPX-O–treated WT mice (*n* = 5 each) and from vehicle-treated (*n* = 7) or CPX-treated (*n* = 8) PKD mice. (**E**) Blood urea nitrogen values measured as mg/dL ± SEM from hemolysis-free serum samples of vehicle- and CPX-O–treated WT mice (*n* = 5 each) or from vehicle- and CPX-O–treated PKD mice (*n* = 5 and *n* = 8, respectively). Statistical significance was determined using unpaired Student’s 2-tailed *t* test (**C**) or 1-way ANOVA followed by Tukey’s HSD test (**D** and **E**) (*P < 0.05, **P < 0.01, ***P < 0.001, and ****P < 0.0001). Scale bar: 1 mm.

We next examined the effects of CPX-O in vivo in a mouse model of ADPKD. We generated *Pkd1^RC/RC^ Pkd2^+/–^* mice (hereafter referred to as PKD mice). These mice show moderate cystic disease progression such that cysts are present at birth and continue to moderately progress until 6 weeks (34). PKD and littermate WT control (*Pkd1^+/+^ Pkd2*^+/+^) mice were weaned at postnatal (P) day 21, and intraperitoneal injections of CPX-O (10 mg/kg body weight) were administered from P22 to P49. Mice were euthanized at P50, and tissues and blood samples were collected (Figure 2A). We did not notice any adverse events with CPX-O treatment at the above dose, and mice remained active throughout the duration of the study. Kidneys of vehicle- and CPX-O–treated WT mice appeared similar. In contrast, a significant reduction in kidney size (Figure 2B) and cystic index (Figure 2C) was observed in PKD mice treated with CPX-O compared with those treated with vehicle. Renal function, as measured by blood urea nitrogen levels, was also statistically different between groups (Figure 2E). As expected, the kidney size correlated with kidney-to-body weight ratio (Figure 2D), showing a significant reduction (*P* < 0.01) in CPX-O–treated PKD group compared with vehicle-treated PKD group. The data clearly showed a protective effect of CPX-O on progression of PKD.

### CPX-O reduces cell proliferation and fibrosis in PKD.

**Figure 3:**
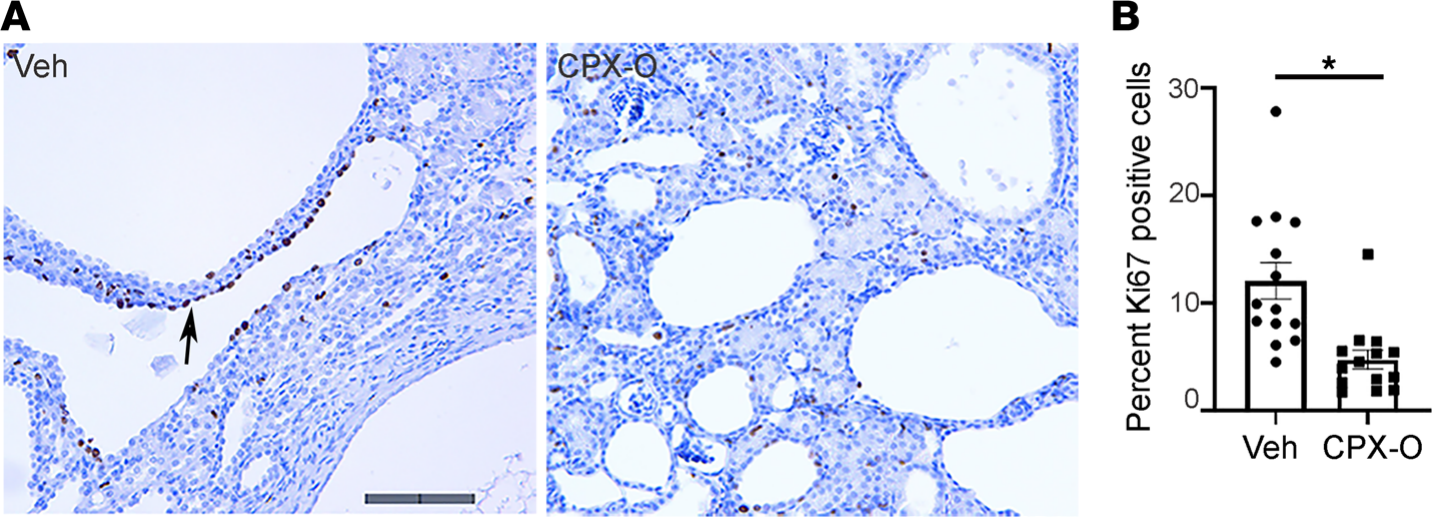
CPX-O slows down cell proliferation in PKD. (**A**) Immunohistochemistry (IHC) for cell proliferation assessed by Ki67 staining on 7-week-old kidneys from PKD mice treated with vehicle or CPX-O. Arrow points to a highly proliferative area on a vehicle-treated (Veh) PKD mouse kidney section. Hematoxylin staining shows nuclei in blue, and Ki67-positive nuclei are shown in dark brown. (**B**) Cells were counted and expressed as percentage Ki67-positive cells ± SEM from at least 14 sections per treatment from 3 mice per group. Unpaired Student’s 2-tailed *t* test used for statistical analysis (*P < 0.05). Scale bar: 100 μm.

To determine whether CPX-O affects cell proliferation and fibrosis in PKD, kidney sections were stained for Ki67 antigen and counterstained with hematoxylin. Ki67-positive cells were clearly seen in abundance in the cystic epithelia (Figure 3A, arrow). Figure 3B represents quantitative analysis where Ki67 labeling is presented as percentage positive cells per section. In addition, Masson’s trichrome staining and α–smooth muscle actin labeling were less intense in CPX-O–treated PKD sections compared with vehicle-treated PKD sections, suggesting a decrease in fibrosis (Supplemental Figure 1, A and B; supplemental material available online with this article; https://doi.org/10.1172/jci.insight.141299DS1).

### CPX-O inhibits ferritin accumulation in the cystic and interstitial compartments in ADPKD kidneys.

**Figure 4:**
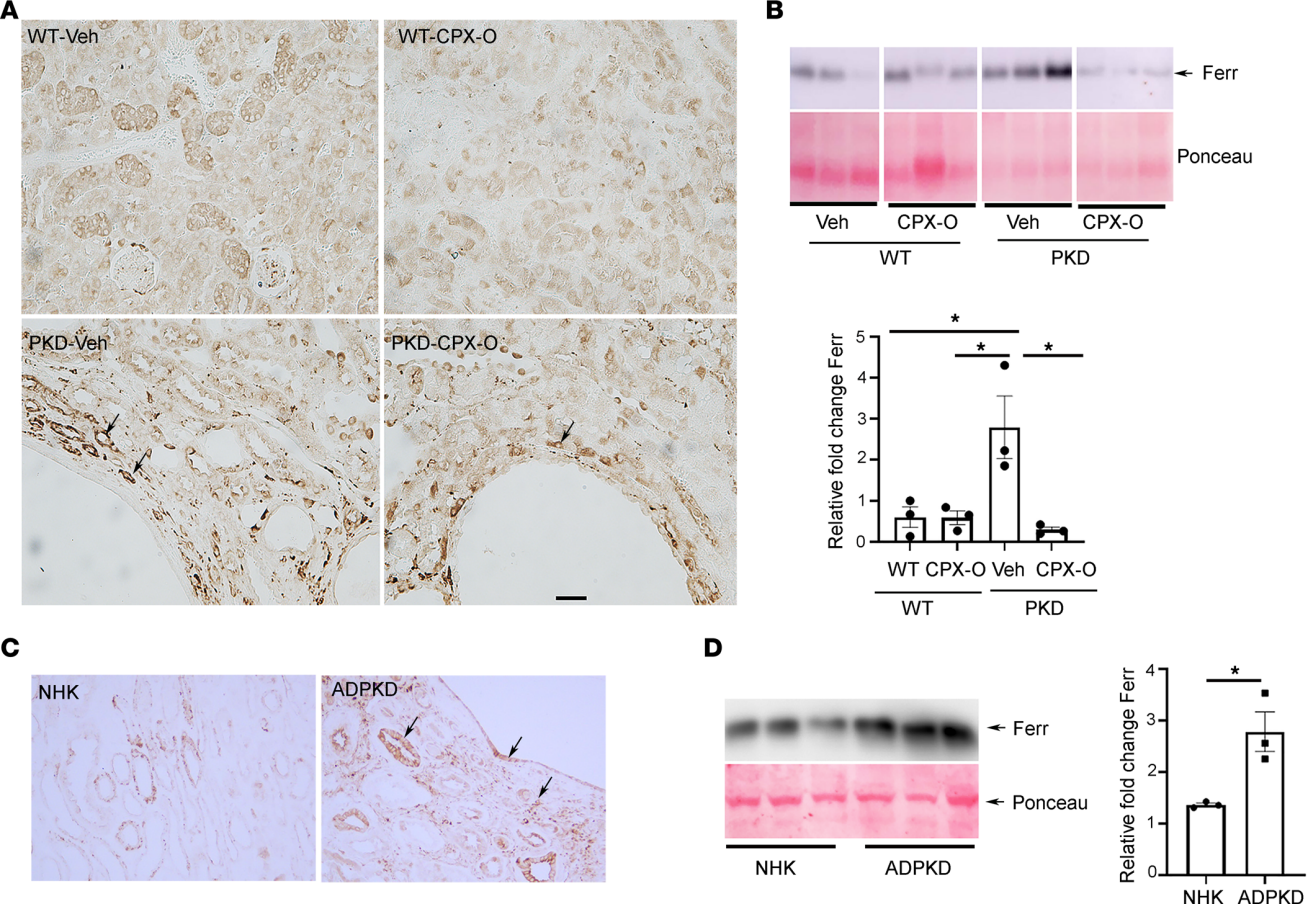
CPX-O inhibits ferritin accumulation in the cystic and interstitial cells in ADPKD kidneys. (**A**) IHC for ferritin in 7-week-old WT and PKD mouse sections treated with vehicle (Veh) or CPX-O. Note (arrowheads) accumulation of ferritin-positive cells near cystic areas in PKD mice. Ferritin-positive cells were reduced in kidneys of CPX-O–treated PKD mice. Scale bar: 100 μm. (**B**) Western blot (WB) of kidney lysates for ferritin (top) and quantification of ferritin expression relative to the Ponceau S expression (lower) in vehicle- and CPX-O–treated PKD mice in contrast to vehicle- and CPX-O–treated WT mice (*n* = 3 per group). (**C**) IHC was performed in NHK and ADPKD kidney for ferritin expression. Arrowheads indicate high ferritin in both cyst epithelium and interstitial cells. Original magnification, ×20. (**D**) WB for ferritin using lysates of primary cells from normal (*n* = 3) or ADPKD (*n* = 3). Quantification of ferritin expression normalized to Ponceau S is shown in the right panel. Data presented as relative fold change in ferritin ± SEM. (Unpaired Student’s 2-tailed t test, *P < 0.05.)

Next, we examined potential mechanisms by which CPX-O could reduce cystogenesis. CPX-O is an iron chelator, and ferritin degradation has been shown as a mechanism of CPX-O (24). Immunohistochemistry for ferritin revealed a marked increase in ferritin expression in kidney sections of vehicle-treated PKD mice versus WT mice. Ferritin was localized in the tubular epithelial cells as well as in the interstitial cells. CPX-O decreased overall ferritin staining in PKD mice and in WT mice as observed by IHC (Figure 4A). Western blot (WB) analysis of vehicle- and CPX-O–treated lysates also showed a significant increase in ferritin expression in lysates of vehicle-treated PKD mice compared with vehicle-treated WT mice (*P* < 0.01). Moreover, ferritin was reduced significantly (*P* < 0.05) in kidney lysates of CPX-O–treated PKD mice (Figure 4B).

**Figure 5:**
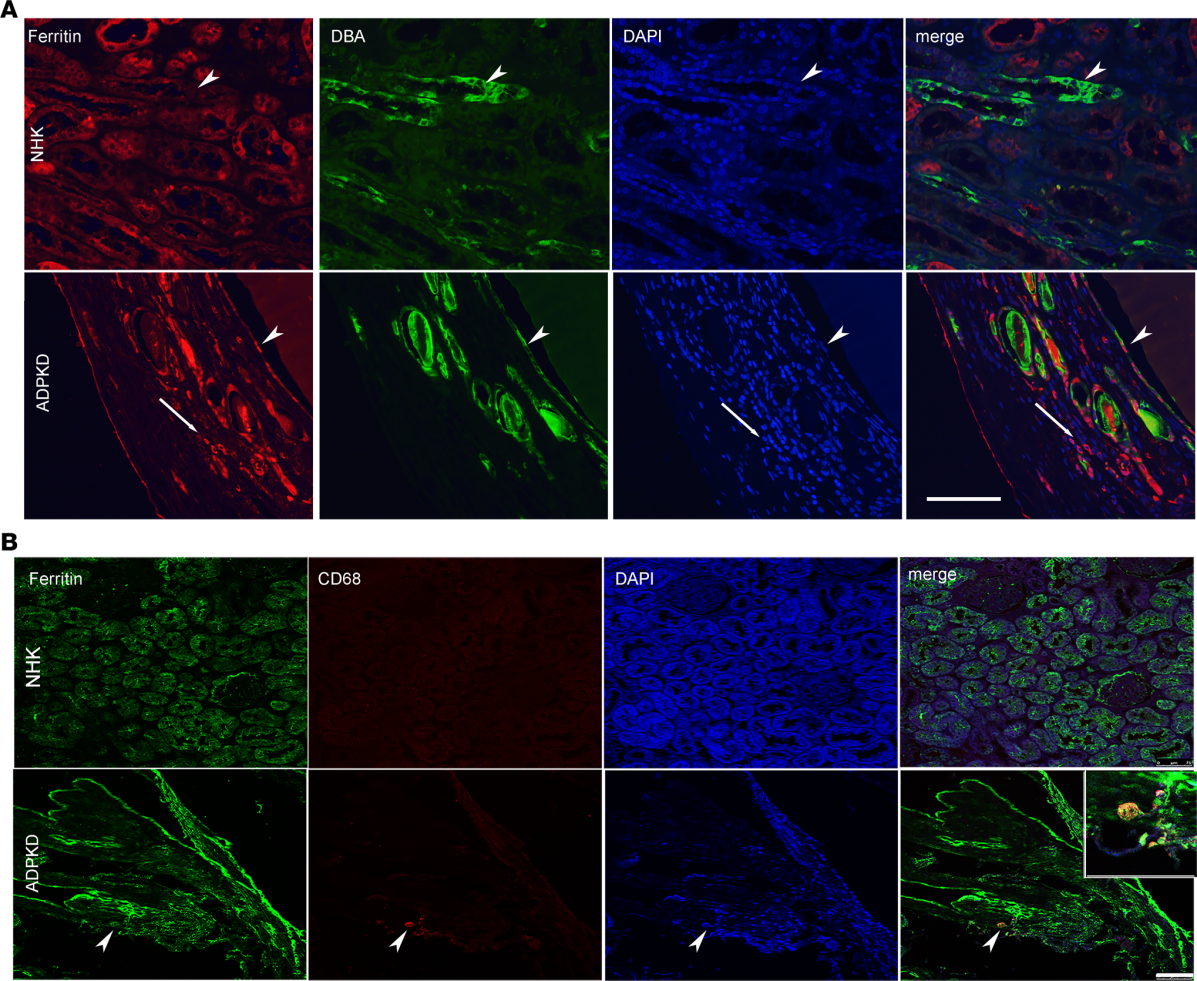
Ferritin is expressed in collecting duct cells and macrophages in patients with ADPKD. (**A**) NHK and ADPKD sections were colabeled for ferritin (red) and DBA (a collecting duct marker, green). DAPI (a nuclear stain, blue) was used as a counterstain. Arrowheads show same cells with coexpression. Merged image from lower panels shows ferritin colocalization with DBA (arrowhead) in cyst-lining cells. Thin arrow shows interstitial cells with ferritin expression. Scale bar: 100 μm. (**B**) NHK and ADPKD sections were colabeled with ferritin (green) and CD68 (macrophage marker) (red). Arrowheads show coexpression of CD68 with ferritin. The area with arrowhead is amplified in the inset (bottom, right panel). Scale bar: 75 μm.

To evaluate ferritin status in patients with ADPKD, we examined ferritin expression in NHKs versus ADPKD kidneys via IHC. Compared with the NHK controls, ferritin expression was elevated in tubular and interstitial compartments of patients with ADPKD (Figure 4C). Further, primary NHK and ADPKD cells (of collecting duct origin) were examined for ferritin expression. As shown in Figure 4D, ferritin was expressed in both NHK and ADPKD cells, but a clear upregulation was observed in ADPKD cells (*P* < 0.05) (*n* = 3). To identify tubules in which ferritin is expressed, NHK and ADPKD kidney sections were stained for ferritin (red) or *Dolichos biflorus* agglutinin (DBA), a marker of collecting ducts (green). Colocalization of DBA and ferritin was observed (Figure 5A, bottom), showing that ferritin is expressed in cysts of collecting duct origin in ADPKD. Ferritin is a ubiquitous protein and has been shown to be localized in proximal tubular cells (35). Consistent with this, ferritin was found expressed normally in proximal tubules in both normal and ADPKD kidneys (Supplemental Figure 2). Some ferritin-positive cells were also observed in the interstitium of ADPKD sections (Figure 5A, arrow). Since ferritin expression has been associated with accumulation of macrophages and macrophages themselves are rich in ferritin (36), we reasoned that these interstitial cells might be macrophages. NHK and ADPKD kidney sections were colabeled for ferritin (green) and a macrophage marker, CD68 (red). As expected, ferritin-positive macrophages were detected in ADPKD kidneys (Figure 5B, inset, lower right).

### CPX-O induces ferritinophagy in an iron-independent manner.

**Figure 6:**
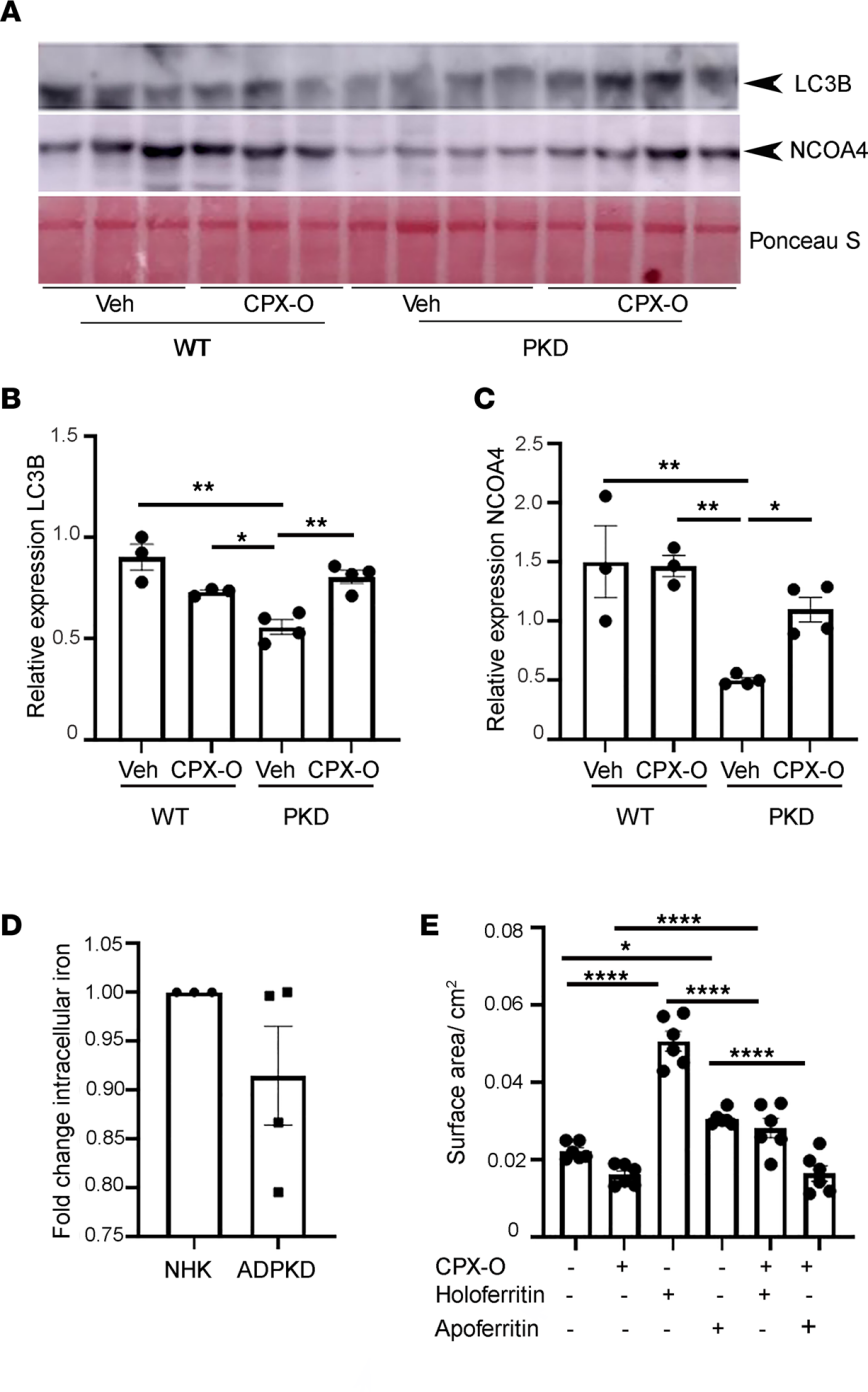
CPX-O induces ferritinophagy in primary cyst epithelial cells from patients with ADPKD. (**A**) WBs for LC3B-II, and NCOA4, on kidney lysates of 7-week-old WT (*n* = 3) and PKD (*n* = 4) mice treated with vehicle (veh) or CPX-O. (**B**) Quantification of LC3B-II from **A** and expression normalized to Ponceau S and expressed as relative expression ± SEM. (**C**) Quantification of NCOA4 expression from **A** normalized to Ponceau S and expressed as relative expression ± SEM. (**D**) Total intracellular iron fold change in ADPKD primary cyst epithelial cells (*n* = 4) relative to NHK primary cells (*n* = 3) ± SEM. (**E**) Cyst-lining epithelial cells from a patient with ADPKD were grown to form cysts on a 3D system using collagen matrix in the presence of FSK and EGF for 5 days followed by treatment with vehicle, CPX-O (5 μM), holoferritin (50 μg/mL), apoferritin (50 μg/mL), holoferritin + CPX-O, and apoferritin + CPX-O for 6 additional days. Cysts were fixed, imaged, and measured. Cyst size is represented as surface area/cm^2^ (*n* = 6/treatment). One-way ANOVA followed by Tukey’s HSD test was used for statistical analyses of all graphs except **D**, where unpaired Student’s 2-tailed *t* test was used (*P < 0.05, **P < 0.01, ****P < 0.0001).

Ferritin degradation via autophagy is one of the proposed mechanisms of action of CPX-O (17). Autophagy is a process that results in the formation of autophagosomes, in which double-membraned vesicular structures sequester cytoplasmic components and fuse with lysosomes to form autophagolysosomes. In these structures, engulfed cargo is broken down by lysosome-derived acid hydrolases. When nuclear receptor coactivator 4 (NCOA4), a cargo receptor, binds to ferritin, it targets ferritin degradation, a process termed “ferritinophagy” (37–41). We determined whether ferritin reduction in CPX-O–treated PKD mice involved autophagy and more specifically ferritinophagy. WB analysis in kidney lysates from mice revealed that an autophagy marker, microtubule-associated protein 1A/1B-light chain 3B-II (LC3B-II), was downregulated in vehicle-treated PKD samples (*n* = 4) relative to vehicle-treated WT samples (*n* = 3) (Figure 6A). Interestingly, CPX-O significantly upregulated LC3B-II levels in PKD kidneys compared with vehicle-treated PKD kidneys (*P* < 0.01), whereas CPX-O had minimal effects on WT kidneys (Figure 6, A and B). When these blots were probed for NCOA4, a specific marker of ferritinophagy, similar results as for LC3B were obtained, showing a clear reduction of NCOA4 expression in vehicle-treated PKD kidneys compared with vehicle-treated WT kidneys (*P* < 0.01). This phenomenon was reversed by CPX-O treatment, resulting in an increase in NCOA4 levels (*P* < 0.05; Figure 6, A and C).

Since CPX-O is a weak iron chelator, we assessed whether CPX-O effects on cyst growth are iron dependent. First, we conducted a colorimetric assay for measuring relative iron levels in NHK (*n* = 3) and ADPKD (*n* = 4) cells. NHK cells and ADPKD cells showed similar iron levels (Figure 6D). This contrasts with ferritin levels, which were consistently increased in ADPKD cells (Figure 4D). Finally, to determine whether ferritin can induce cyst growth and whether CPX is dependent on iron to slow cyst growth, ADPKD cells were grown in 3D culture to form cysts, followed by treatment with CPX-O, holoferritin (ferritin with iron), apoferritin (ferritin without iron), or a combination. Holoferritin and apoferritin alone induced cyst growth (Figure 6E). Compared with apoferritin, holoferritin-induced cysts were larger (*P* < 0.0001). However, CPX together with either holoferritin or apoferritin reduced cyst growth (*P* < 0.0001). Finally, we asked whether a commonly used clinical iron chelator such as deferoxamine (DFO) would achieve similar effects as CPX-O. We treated WT and PKD mice with DFO (100 mg/kg body weight) for 27 days. This dose was previously used to treat vascular inflammation in mice (42). Kidneys were extracted and evaluated for disease. No changes in the kidneys of PKD mice were observed, and renal cystic index measurements were similar between vehicle- and DFO-treated PKD mice (Supplemental Figure 3). Taken together, the data indicate that ferritin can induce cyst formation and that CPX acts in an iron-independent manner to slow cyst progression.

## Discussion

In recent years, CPX or CPX-O has emerged as an important anticancer agent (23). Although CPX is considered a weak iron chelator, both iron-dependent and -independent mechanisms of action of CPX have been reported (13). In a recent study, CPX-O was used against congenital erythropoietic porphyria, a disease characterized by marked accumulation of uroporphyrin 1 (URO1). CPX reduced the URO1 levels in an iron-independent manner (43). In another study, the effects of CPX on proliferation of a neuroblastoma cell line, CHP134, were evaluated (44). CPX reduced proliferation of these cells; however, adding iron to the media only partially prevented antiproliferative effects of CPX, showing iron-independent effects of CPX. We found that CPX-O treatments in mice prevented cyst progression in PKD mice, but treatments with DFO did not. These results indicate that CPX-O and DFO work in distinct ways. One explanation for these results is that CPX-O targets intracellular iron while DFO targets only the extracellular iron as has been reported (44, 45). Another explanation is that CPX-O may work independent of iron.

Iron is an essential element in the body that is ubiquitously distributed and regulates the heme pathway and several enzymes in the body that carry out physiologic functions. Unbound intracellular iron generates ROS through Fenton chemistry, leading to DNA breaks, lipid peroxidation, and cellular damage. Iron regulates ferritin, which is the major iron-storing protein in mammalian cells. The composition of ferritin in different tissues is based on their iron requirements. Heavy ferritin (*FTH1*) is abundantly found in brain, muscles, heart, and kidney while light ferritin (*FTL*) is rich in spleen and liver (35, 46). Ferritin expression is regulated transcriptionally by an iron-responsive element (IRE) located within the untranslated regions (UTRs) of the mRNA. Depending on iron requirements, iron regulatory protein is activated, binds to the IRE in the 5′ UTR of iron transport proteins, and either degrades or stabilizes their mRNA so that the expression of ferritin can be regulated. Ferritin expression can thus be increased or decreased based on the requirement of iron in the cell (46). However, noncanonical regulation of ferritin is now well recognized, especially in cancer, where ferritin expression can be regulated by inflammation, oxidative stress, and hypoxia (46). Moreover, iron-independent roles of ferritin have been proposed (47, 48). High ferritin expression can enhance cell growth, and its downregulation can disrupt the tumor microenvironment (49). In our 3D in vitro cyst growth assays, we found that holoferritin induced a substantial growth of cysts. Apoferritin also induced cyst growth, albeit less than holoferritin, showing that the presence of iron can induce cyst growth. However, CPX repressed both holoferritin- and apoferritin-induced cysts.

ADPKD cells used in our study are derived from unrelated ADPKD patients of differing genetic backgrounds. Moreover, depending on the mutations these patients carry, variable data can be obtained. While we consistently found increased ferritin in all patients, iron levels were variable and nonsignificant in cells obtained from ADPKD compared with normal kidneys. This reflects an imbalance of the iron/ferritin axis in PKD. The effect of CPX-O on ADPKD cells was also variable. In most cells, doses as low as 0.5 μM CPX-O were effective (Figure 1), and very rarely, 5 μM CPX-O was used to counter the cyst-promoting effects of holoferritin and apoferritin (Figure 6). In both cases, ADPKD cells were more responsive to CPX-O than NHK cells. This is in contrast to a range from 0.5 μM to 10 μM used in various cancer cell lines, such as HPV-positive cancer cells, pancreatic cancer cell lines, and neuroblastoma cell lines (21, 50, 51). Moreover, we used CPX-O at a concentration of 10 mg/kg body weight for 27 days, whereas others have used the range of 20–60 mg/kg to observe therapeutic effects in mouse models of pancreatic cancer and diabetes (50, 52).

To study the effects of CPX-O on ferritin in the context of PKD, we evaluated ferritin levels in PKD. Corroborating previous reports, we demonstrate that ferritin is expressed in the proximal tubules of the kidney (35). However, we found that primary cyst epithelial cells from ADPKD patients, which are of collecting duct origin, showed high levels of ferritin. Also, DBA-positive cells in PKD kidneys stained positive for ferritin, indicating that under pathologic circumstances, ferritin is increased in cyst-lining cells, similar to that reported in cancer and other pathologic diseases (36, 48, 53–55). We also observed the presence of ferritin in interstitial cells, some of which were identified as macrophages. In fact, it was reported that macrophages may transfer ferritin to oligodendrocytes (NG2) in the spinal cord. This resulted in migration and proliferation of NG2 cells (56). These data together with the antifibrosis effect of CPX-O indicate that CPX-O may also target ferritin in immunologic cells in the kidney. Previous observation has shown that active hepatic stellate cells in liver injury express ferritin, which activates NF-κB and plays a proinflammatory role. These effects of ferritin were shown to be iron independent (47). Based on this, we speculate that cyst epithelial cells may express ferritin and promote inflammation in the cystic milieu, and this inflammation in turn may promote ferritin expression in the cyst epithelial cells. However, to precisely evaluate the pathogenic role of ferritin in cystogenesis, targeted manipulation of ferritin in collecting ducts is warranted.

Conditional knockdown of proximal tubule–specific FTH1 in mice worsened acute kidney injury and was associated with increased apoptosis and significant mortality. The study indicated a protective role of FTH1 in proximal tubules in acute kidney injury (35). On the contrary, deletion of myeloid FTH1 was protective against lipopolysaccharide-induced endotoxemia in mice (57).

In breast cancer cells, binding and uptake of ferritin were observed along with increased cell growth, an effect that was independent of the iron status of ferritin (48). Ferritin promotes angiogenesis by activating ERK and AKT signaling in endothelial cells (54). These prosurvival pathways are also activated in ADPKD cells and associated with increased cell proliferation (3, 58). In our study, CPX-O treatments reduced cell proliferation in PKD kidneys as evident from Ki67 staining. Thus, ferritin is also likely involved in proliferation of ADPKD cells. Whether ferritin is involved in increased fluid secretion remains to be determined.

Renal expression of autophagic marker (LC3B-II) was decreased in PKD mice. Reduced autophagy has been reported in PKD mice and induction of autophagy attenuated disease (59, 60). In our study CPX-O induced autophagy, specifically the autophagy of ferritin as revealed by NCOA4 expression with LC3B expression data. Taken together, these findings suggest that specific autophagic ferritin degradation (ferritinophagy) is a mechanism of CPX-O action in PKD kidneys. Ferritinophagy also has been reported to be a key mechanism maintaining iron homeostasis (41, 61).

Thus far, the role of iron-ferritin metabolism in PKD has been elusive, and to our knowledge this is the first demonstration that ferritin is dysregulated in cystic cells and that CPX-O can be a potential therapy for PKD. Available literature on extracellular ferritin studies shows that patients with ADPKD have higher levels of hemoglobin compared with patients with other forms of chronic kidney disease. This has been thought to be a consequence of increased erythropoietin production by cystic cells (62, 63). In our hands, iron levels in NHK and ADPKD cells did not differ in vitro. It has also been shown that average ferritin levels of 100–800 ng/mL were associated with the best survival in patients with PKD, whereas that of non-PKD patients was 500–800 ng/mL (64). The study opens avenues for future investigations regarding the role of iron and ferritin homeostasis in PKD.

## Methods

### Antibodies.

Antibodies, catalog numbers, and their sources are listed: LC3B (ab51520), CD68 (ab125212), smooth muscle actin (ab5694), ferritin (ab75973) (Abcam), NCOA4 (SAB1409837) (MilliporeSigma), anti-mouse IgG peroxidase conjugated (W402B, Promega), anti-rabbit IgG peroxidase conjugated (ZE 0614, Vector Laboratories), goat anti-rabbit IgG Alexa Fluor 488 (ab150077) and goat anti-mouse IgG Alexa Fluor 594 (ab150116) (Abcam), and horse anti-mouse biotinylated antibody (BA-2000) and goat anti-rabbit biotinylated antibody (BA-1000) (Vector Laboratories).

### Reagents.

We used DAPT (APExBIO); ciclopirox olamine, holoferritin, apoferritin, and iron assay kit (MilliporeSigma); and DBA and *Lotus tetragonolobus* lectin (Vector Laboratories).

### Animal care and protocol.

*Pkd1^RC/RC^ Pkd2^+/–^* (PKD) mice were generated by breeding *Pkd1^RC/RC^* mice with *Pkd2^+/–^* mice (both in C57BL/6J background) provided by Peter Harris (Department of Biochemistry and Molecular Biology, Mayo Graduate School of Biomedical Sciences, Rochester, Minnesota, USA) and Steven Somlo (Section of Nephrology, Department of Internal Medicine, Yale University School of Medicine, New Haven, Connecticut, USA), respectively (65, 66). Homozygous *Pkd1^RC/RC^* mice have slowly progressing cyst phenotype whereas heterozygous *Pkd2^+/–^* mice have no cyst phenotype. However, when these mice are bred to obtain *Pkd1^RC/RC^* *Pkd2^+/–^* mice, cyst formation is accelerated. WT littermates without a PKD mutation (*Pkd1^+/+^ Pkd2*^+/+^) were used as controls. The *Pdk1^RC/RC^* *Pkd2^+/–^* (PKD) mice are mildly cystic at birth, and the cysts progress with age such that there is an exponential growth of the cysts between age P15 and P60, after which cyst growth does not advance (34). We used these mice because the time period of cyst formation was moderate for the drug studies. Mice were obtained from the PKD Rodent Model and Drug-Testing Core at the University of Kansas Medical Center.

### CPX-O and DFO treatments in mice.

CPX-O was solubilized in 0.5 M phosphate buffer pH 9.5 and filter sterilized using a 0.2 μm filter for in vivo treatments. Study groups consisted of (a) vehicle-treated WT mice, (b) CPX-O–treated WT mice, (c) vehicle-treated PKD mice, and (d) CPX-O–treated PKD mice. Mice were weaned and treatments were started at P22 for a total of 27 days with daily intraperitoneal injections of 10 mg/kg body weight CPX-O (Figure 2). During euthanasia, mice were weighed and perfused with cold PBS after blood collection followed by collecting and weighing kidneys. One kidney was snap-frozen and the other was fixed in 4% paraformaldehyde for 24 hours followed by storage in 70% ethanol at 4°C until blocking and sectioning for histology and IHC. Similarly, for DFO treatments, DFO was solubilized in water (100 mg/kg body weight) and intraperitoneally injected into mice for 27 consecutive days followed by euthanasia and sample collection.

### Histology, cystic index, and blood urea nitrogen measurements.

The fixed kidney tissues were processed and embedded in paraffin at the core facilities of the University of Kansas Medical Center (KUMC). Five-micrometer sections were stained with H&E as described previously (67). Cystic index was measured using ImageJ (NIH)on H&E-stained kidney sections. The area of each individual cyst within the section of the entire kidney was calculated and then added together. This summed value was then divided by the total area of the section yielding the value identified as the cystic index. This was done for kidney sections for every vehicle- or CPX-O–treated PKD mouse used in the study. Blood urea nitrogen (BUN) was quantified using QuantiChrom Urea Assay Kit (BioAssay Systems), according to the manufacturer’s protocol. For BUN, only hemolysis-free serum samples were used.

### Human cells and tissues.

ADPKD kidneys were obtained from the surgery department at KUMC with the assistance of the KU Cancer Center’s Biospecimen Repository Core Facility and hospitals participating in the tissue donation program at the PKD Foundation (Kansas City, Missouri, USA). NHK tissues were obtained from nephrectomy specimens taken by the surgery department at KUMC. Primary cultures of ADPKD and NHK epithelial cells were generated as described previously (6). Use of deidentified surgically discarded tissues complied with federal regulations and was approved by the Institutional Review Board at KUMC. ADPKD cells were obtained from multiple surface cysts ranging in size. NHK cells were cultured from sections of cortex. These cells have been shown to be enriched in collecting duct marker DBA (3). Cells were cultured in DMEM/F12 supplemented with 5% FBS; 5 μg/mL insulin, 5 μg/mL transferrin, and 5 ng/mL sodium selenite (ITS, Thermo Fisher Scientific); and penicillin (100 U/mL) and streptomycin (130 μg/mL) (68). Cultures were not passaged more than twice before being used in experiments. Cells were grown in an incubator at 37°C under 5% CO_2_.

### Cell culture and treatments.

NHK cells and ADPKD cells were grown to 80% confluence in a 10 mm cell culture dish. Cells were washed and lysed with RIPA lysis buffer (50 mM Tris-HCl pH 7.5, 137 mM NaCl, 1% IGEPAL, 2 mM EDTA) and Complete Protease Inhibitor (Thermo Fisher Scientific) for WBs. For treatments, ADPKD cells were grown to 70% confluence followed by a 24-hour low-serum (0.001% and no ITS) treatment. Cells were then treated with vehicle or CPX-O for 24 hours before lysis.

### Cell viability assay.

ADPKD or NHK cells were plated in a 12-well plate (20,000 cells/well) and allowed to grow overnight. The following morning cells were treated with either vehicle control (0.5 M phosphate buffer pH 9.5) or increasing concentrations of CPX-O. Cells were grown for 6 days, and fresh medium containing CPX-O was replaced every day. After 6 days, cells were trypsinized and pelleted. Cells were suspended in 500 μL media, cell viability was tested in triplicates using Cell Counting Kit-8 (APExBIO), and manufacturer’s instructions were followed. Viability was set at 100% for the vehicle control, and relative values were calculated for other doses.

### In vitro 3D cyst assays.

In vitro cyst assays were performed as described (6, 33, 69). Briefly, primary cultures of ADPKD cells were suspended in media containing type I collagen (PureCol, Advanced Biomatrix) in a 96-well plate. Immediately after adding collagen and cells (4 × 10^3^/100 μL), 100 μL of media with collagen and cells was pipetted into each well of the 96-well plate. The plate was incubated at 37°C for 45 minutes to allow collagen to polymerize. Then, 150 μL of defined media (1:1 DMEM/F12 with ITS, 5 × 10^−8^ M hydrocortisone, 5 × 10^−5^ M triiodothyronine) containing 5 μM FSK and 5 ng/mL EGF was added onto the polymerized gel to initiate cyst growth. Following cyst growth between day 5 and 6, the agonists (FSK and EGF) were removed, and the gels were rinsed twice with defined media. To initiate drug treatments, CPX-O at different concentrations was added to the wells. For control, media containing 50 μM DAPT was added. In other experiments, holoferritin or apoferritin (50 μg/mL) was added in the culture media with or without CPX-O (5 μM). Fresh treatment medium was replaced every day for each treatment. After 5–7 days, the outer diameter of cross-sectional images of spherical cysts with distinct lumens were measured using a digital camera attached to an inverted microscope and analyzed with video analysis software (Image-Pro Premier, Media Cybernetics). Surface area was calculated from the outer diameters, and total surface area of the cysts was determined from the sum of individual cysts within each well. Cysts with diameters of 50 μM or less were excluded. Data are presented as surface area/μm^2^ or surface area fold change. Experiments were replicated 6 times from at least 3 ADPKD patients or presented as an average of 6 replicates from a single patient sample.

### IHC/immunofluorescence.

IHC was performed as described previously (70). Briefly, kidney sections from WT and PKD mice treated with CPX-O or vehicle (veh) were deparaffinized with xylene and hydrated with graded ethanol. Sections were then boiled in citrate buffer (10 mM sodium citrate, 0.05% Tween 20, pH: 6.0) and cooled to room temperature. Sections were incubated for 30 minutes with 3% hydrogen peroxide for IHC or 0.5 M ammonium chloride for immunofluorescence (IF) to block endogenous peroxidase or fluorescence activity, respectively. Subsequent washing in PBS and blocking with 10% normal serum (in PBS from the species the secondary antibody was raised in) for 1 hour were followed by incubation for 1 hour with primary antibodies in a humidified chamber. Slides were washed 3 times in PBS and incubated for 1 hour in 1:400 diluted biotin-conjugated secondary antibodies for IHC and 1:400 Alexa Fluor 488 or Alexa Fluor 594 antibodies for IF. Slides were washed 4 times in PBS. For IF, the slides were coverslipped using VECTASHIELD with DAPI (Vector Laboratories). For IHC, the slides were further incubated with avidin-biotin-peroxidase complex (ABC Elite; Vector Laboratories) and detected with diaminobenzidine (MilliporeSigma). Tissue sections for IHC were then dehydrated with graded ethanol and mounted with Permount (Thermo Fisher Scientific). Slides were viewed on a Leica Microsystems TCS SPE II or a Leica Microsystems SP8 confocal microscope.

For quantification of cell proliferation, Ki67-labeled sections were counterstained with hematoxylin to visualize nuclei. Images were acquired from 4 random fields from each mouse section (total 4 mice each group), and total numbers of nuclei and Ki67-positive cells were counted in a blinded fashion. Data from each field were averaged and presented as percentage Ki67-positive cells.

### Western blots.

Following treatments, cells were washed with PBS 3 times and lysed. Tissues (fresh or frozen) were chopped in pieces and homogenized using a Dounce homogenizer. For both cells and tissues, RIPA lysis buffer (50 mM Tris-HCl pH 7.5, 137 mM NaCl, 1% IGEPAL, 2 mM EDTA) with protease inhibitors (Protease Inhibitor Cocktail, Thermo Fisher Scientific) was used (8). Protein concentration was measured using BCA protein assay (Bio-Rad). Whole cell lysates (50 to 100 μg) were electrophoresed on 15% (for ferritin and LC3B) and 10% (for other proteins) polyacrylamide gels. Proteins were transferred to PVDF membranes. Ponceau S staining was performed for each blot to determine protein transfer and imaged. Ponceau S was also used to normalize the gels for protein loading (71). The immunoblots were blocked in 5% nonfat dry milk in PBS containing 0.1% Tween 20 (PBST) for 1 hour at room temperature followed by PBS washes; the blots were incubated with appropriate dilutions of primary antibodies overnight. The blots were then washed and incubated with secondary antibodies (1:10,000 dilution in blocking solution) for 1 hour at room temperature. After subsequent washes in PBST, bound antibody was detected by chemiluminescence (Western Lightning Plus ECL, PerkinElmer). Bands produced in the results were quantified using ImageJ and normalized with Ponceau S staining to confirm equal loading (71). Data were presented as relative fold change.

### Iron measurements.

NHK and ADPKD cells were grown to 70% confluence followed by trypsinization. Iron assays were done in 3 × 10^6^ cells per patient sample. Iron was measured using colorimetric kit (MilliporeSigma) using manufacturer’s instructions. Data are presented as relative fold change.

### Statistics.

Data are expressed as mean ± SEM. Statistical significance was measured by Student’s unpaired 2-tailed *t* test for comparison between control and PKD groups. One-way ANOVA was performed to compare more than 2 groups followed by Tukey’s HSD test. A *P* < 0.05 was considered statistically significant.

### Study approval.

All mice were maintained in accordance with the recommendations in the *Guide for the Care and Use of Laboratory Animals* of the NIH (National Academies Press, 2011). The animal experimental protocol was approved by the KUMC Institutional Animal Care and Use Committee. Primary ADPKD and NHK cells and fixed tissues were obtained from the Biomarkers, Biomaterials, and Cellular Models Core in the Kansas PKD Center at KUMC. The use of discarded human tissues for research complies with federal regulations and was approved by the Institutional Review Board at KUMC.

## Author contributions

MS conceived and designed the study. MS, PSR, MMT, JDP, RVP, SY, and BM conducted experiments; HZ, PVT, and DPW provided reagents; MS, DS, SB, HZ, PVT, DPW, and JPC analyzed data and interpreted results of experiments; MS prepared figures and drafted the manuscript; MS, SB, HZ, DS, PVT, DPW, and JPC edited and revised the manuscript; and MS approved the final version of the manuscript. All authors read and approved the final manuscript.

## Supplementary Material

Supplemental data

## Version 1 03/30/2021

In-Press Preview

## Version 2 04/22/2021

Electronic publication
