# Supplementary material for: Ciclopirox olamine induces ferritinophagy and reduces cyst burden in polycystic kidney disease
Source: JCI Insight. 2021 Mar 30;6(8):e141299. doi: 10.1172/jci.insight.141299 (PMC8119220; doi:10.1172/jci.insight.141299)
Supplement: Supplemental data [file jciinsight-6-141299-s007.pdf]

Supplementary figure 1

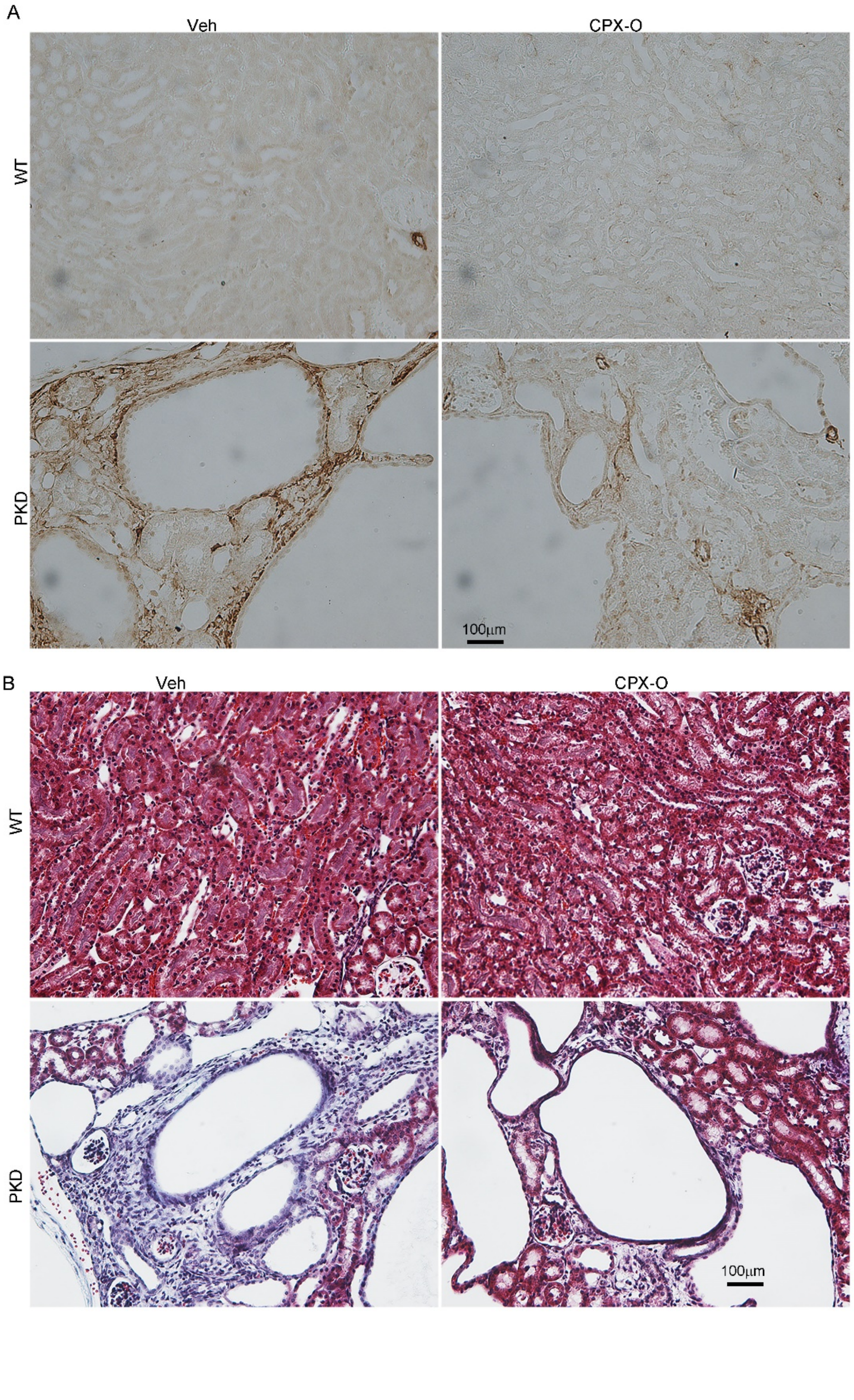

CPX reduces overall renal fibrotic areas in PKD mice: (A) Immunohistochemistry for detection of smooth muscle actin shows increased labeling in fibrous tissue surrounding the smooth muscle cells in PKD mouse sections, treatment with CPX-O treated with CPX-O reduced these effects. **(B)** Mason Trichrome staining detecting collagenous stroma and myofibroblastic cells (blue colored) in renal tissues of PKD mice treated with vehicle or CPX-O .

Supplementary figure 2

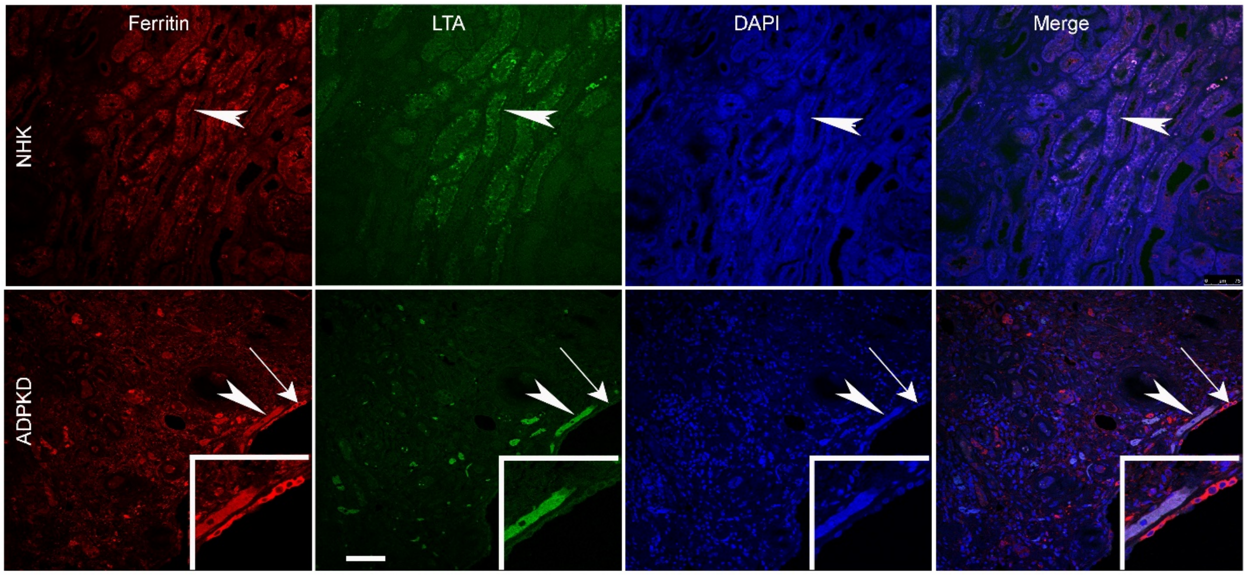

Ferritin is expressed in proximal tubules in both NHK and ADPKD patients: (A) NHK (upper panel) ADPKD sections (lower panel) were co-labelled for ferritin (red) and LTA (a proximal tubule marker, green), DAPI (a nuclear stain, blue) was used as a counterstain. Merged images from right panels show ferritin co-localization with LTA (pink). Arrowheads show LTA positive tubule segment where ferritin is expressed. Thin

arrows show cyst lining epithelia highly positive for ferritin (red). This area has been amplified in the inset on lower panels. Scale bar: 75μm.

Supplementary figure 3

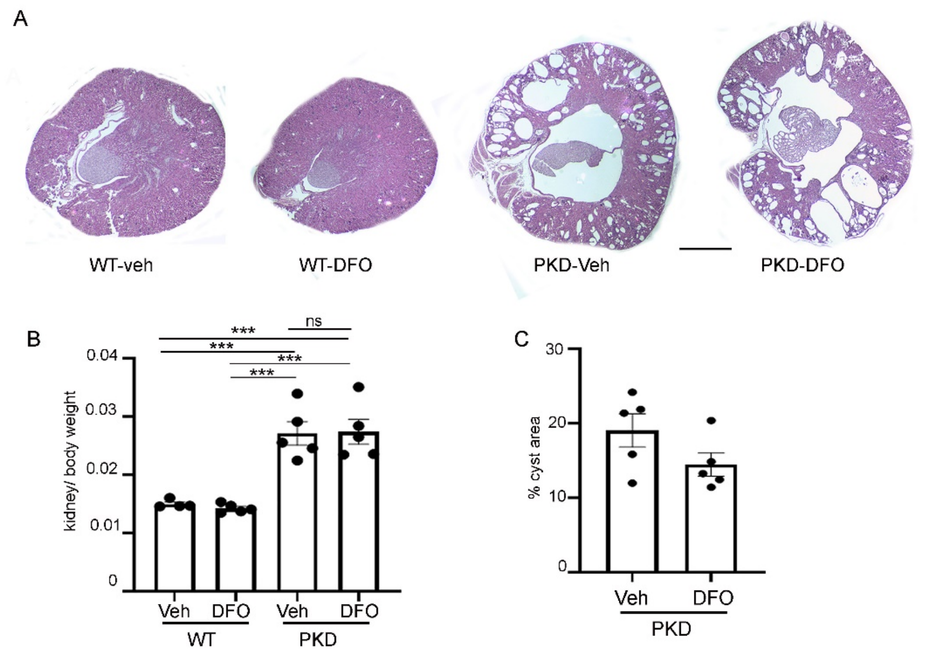

DFO does not alter disease progression in a mouse model of ADPKD: (A) PKD mice (n=5) or WT mice (n=5) were intraperitoneally injected with vehicle (Veh) or DFP (100mg/kg body weight) for 27 consecutive days. The following day, mice were euthanized and kidneys were collected and fixed for H & E staining. Representative images of kidney sections from each treatment group are shown. **(B)** Renal cystic index of vehicle and DFO treated PKD mice, measured as percent cystic area. Kidney weight to body weight ratio. Data presented as mean ± SE. Statistical significance was determined using unpaired student's T-test (C) or one-way ANOVA followed by Tukey's HSD test (B) (*p<0.05). Scale bar: 1mm.
